# Visual‐Somatosensory Integration: A novel behavioral marker of preclinical Alzheimer’s disease?

**DOI:** 10.1002/alz.087472

**Published:** 2025-01-09

**Authors:** Jeannette R Mahoney, Emmeline Ayers, Joe Verghese

**Affiliations:** ^1^ Albert Einstein College of Medicine, Bronx, NY USA

## Abstract

**Background:**

Growing evidence suggests that Alzheimer’s pathology manifests in sensory association areas well before appearing in neural regions involved in higher‐order cognitive functions, such as memory. The ability to successfully integrate sensory information across multiple sensory modalities is a vital aspect of everyday functioning and mobility. Our research suggests that multisensory integration, specifically visual‐somatosensory integration (VSI), could be used as a novel marker for preclinical AD given previously reported associations with important motor (balance, gait, and falls) and cognitive (attention) outcomes in aging.

**Method:**

243 older adults (77 ± 6.5yrs; 52% female) with and without cognitive impairments that provided blood (plasma) specimens for amyloid‐beta (Aβ) testing were included in this cross‐sectional observational study. Participants completed a multisensory simple reaction time test in response to visual, somatosensory, and combined visual‐somatosensory stimulation. Magnitude of VSI was computed and served as the independent variable, while Aβ probability scores served as the dependent variable.

**Result:**

Linear regression revealed a significant association between magnitude of VSI and Aβ probability scores, even after controlling for several key covariates (β = ‐.17; p <.01). Results indicate an inverse association of Aβ probability with magnitude of VSI, where increased Aβ probability scores were associated with worse multisensory integration performance.

**Conclusion:**

These findings provide support for VSI as a potential novel non‐cognitive, non‐invasive biomarker of preclinical Alzheimer’s disease.

